# Treatment of the Nerves of the Teeth

**Published:** 1840

**Authors:** E. Baker


### ON THE TREATMENT OF THE NERVES OF THE TEETH.

[ BY E. BAKER. ]

In the last number of this journal, there is an extract from an article of Mr. Koecker's, wherein he described his method of treating the nerves of the teeth when exposed.

Preceding the "author's method," there is an invitation, for correspondents to furnish an article on the same subject, "invalidating or confirming" the doctrines therein advanced.

It is customary, in medical journals, for the editor to comment on the practice contained in the articles sent in for publication. Nothing can be more reasonable and necessary than this. No principles or practice should be introduced, calculated to mislead, or have an injurious effect, without being accompanied with such remarks as may be suggested, either in favor, or at any rate, when he sees reason for an opposite opinion ; and it must be of the utmost importance, that no article be admitted into the Journal of Dental Science, without the suitable accompanying remarks of the editors—rendered the more necessary, as both the principles and practice of dentists, in the mass, are so very conflicting. That the editors of this journal are amply qualified to discharge this important duty, cannot be disputed—and their omission in performing it, is probably to be ascribed to motives of delicacy, which, as editors, "would be better manifested in the breach than in the observance."

It is only necessary to recapitulate so much of Mr. Koecker's method of operating, in the case of exposed nerves, as will answer our purpose. He begins with giving a description of the instrument he makes use of, together with the manner of operating, viz : by touching the exposed part of the *membrane* with a red hot wire, &c. Now does Mr. K. mean the nerve of the tooth or not? A membrane is represented by the physiologist, as a thin expanded substance, composed of cellular texture, viz : such as the skin, dura mater, periosteum, &c. It is supposed, however, he means the *nerve*, as he says, "he touches the exposed part very rapidly, so that its surface contracts, without, however suffering it to penetrate deeply into the nerve." But he still goes on to say, that this penetrating deeply into the *nerve* "would inevitably bring on suppuration and destruction of the whole lining *membiane*," by which, we understand periosteum, &c. "Having perfectly dried the cavity," he now takes a small plate of very thin leaf lead, and lays it on the exposed nerve, and on the immediate surrounding bony parts, and then carefully fills up the whole cavity with gold.

We are to infer, (though not expressly informed) that Mr. K. performs this operation, with at least, considerable success. Perhaps Mr. K. from his undoubted skill, has been vastly more successful in this operation than others. From my great respect for Mr. K's opinions and practice, I perseveringly tried his method of treating an exposed nerve, but have long since abandoned it, not meeting with the success that would, by any means warrant the continuance of the practice; and I am not informed that any of my professional brethren have successfully adopted it. It appears that the nerve of a tooth is so highly organized that it will not admit of being wounded, and frequently, not of a near approach, without being either immediately or mediately destroyed: sometimes by a violent inflammation, and then again, by little or none. The consequence is, what is usually called a gum boil succeeds, and then unless the after treatment is proper, the disease often increases to such a degree as to become a nuisance, and ultimately the tooth is lost.

After having assailed the practice of one so eminent as Mr. K. it may be expected the writer will state what he considers a better method of treatment in similar cases, and hopes to escape censure, as the necessity of correct professional practice, will not be disputed by any one.

When the nerve of a front tooth, or the nerve of one of the bicuspides, is from the depth of the decay, unavoidably exposed, it is less objectionable, and I believe, is the practice adopted by the most successful dentists at this time, to proceed to destroy the nerve directly, by running a small instrument up the cavity of the tooth as high as where the nerve commences to go through the foramen at the end of the root. The instrument there meeting with resistance from the diminished size of the canal, cuts off the nerve at that point. The nerve being taken out the internal cavity after having been made perfectly clean and dry, should be immediately filled with gold to its highest point. There is seldom little, and often, no inflammation in a tooth after being treated in this manner, and what is of very great importance, there is very seldom a gum boil, provided the operation has been performed with competent skill. The late Mr. Hudson of Philadelphia, who certainly has not been excelled, if equalled, as an operator, followed this practice more than thirty years since, and teeth treated by him in this manner, remain to this time.

Gum boils have been incidently mentioned in this communication, an account of the generally successful treatment of teeth affected with this disease, would be interesting, but uncalled for at this time.

